# Adherence to Healthy Lifestyle Habits Is a Determinant of the Effectiveness of Weight Loss among Patients Undergoing Endoscopic Bariatric Therapies

**DOI:** 10.3390/nu14112261

**Published:** 2022-05-28

**Authors:** Gemma Miranda-Peñarroya, María Fernanda Zerón-Rugerio, Marta Vallejo-Gracia, Ricardo Sorio-Fuentes, Fernando Saenger-Ruiz, Maria Izquierdo-Pulido

**Affiliations:** 1 Clínica Opción Médica S.L., C. Calvet 24, 08021 Barcelona, Barcelona, Spain; gemmamiranda1971@gmail.com (G.M.-P.); martavallejogracia@gmail.com (M.V.-G.); dr.sorio@opcionmedica.com (R.S.-F.); dr.saenger@opcionmedica.com (F.S.-R.); 2 Departament d’Infermeria Fonamental i Medicoquirúrgica de la Facultat de Medicina, Universitat de Barcelona, Feixa Llarga, 08907 l’Hospitalet de Llobregat, Barcelona, Spain; 3 Departament de Nutrició, Ciències de l’Alimentació i Gastronomía, Campus de l’Alimentació Torribera, Universitat de Barcelona, Av. Prat de la Riba 171, 08921 Santa Coloma de Gramenet, Barcelona, Spain; fernanda.zeron@ub.edu; 4 Institut de Recerca i Seguretat Alimentària (INSA-UB), Universitat de Barcelona, Av. Prat de la Riba 171, 08921 Santa Coloma de Gramenet, Barcelona, Spain

**Keywords:** obesity, endoscopic bariatric therapy, healthy lifestyle habits, diet, physical activity, weight loss effectiveness, emotional eating, psychological traits

## Abstract

Endoscopic bariatric therapies (EBTs) are promising strategies for the treatment of obesity. However, there is still great variability in its effectiveness in weight loss. Thus, we investigated whether adherence to healthy lifestyle habits is a significant determinant of weight loss effectiveness among patients undergoing EBTs. Additionally, the role of eating behaviors and psychological traits in the effectiveness of weight loss was studied. A cohort of 361 participants (81.4% women; age 41.8 ± 9.5 years; BMI 37.8 ± 4.3 kg/m^2^) was followed for 1 year after EBT. Anthropometric parameters, adherence to healthy lifestyle habits, emotional eating, and psychological traits (anxiety and depression) were evaluated. General linear models were used to compare outcome variables according to weight loss effectiveness groups (poor vs. good weight-loss-responders). Additionally, a hierarchical linear regression model was used to determine whether adherence to healthy lifestyle habits, emotional eating, or psychological traits were significant predictors of excess weight loss (%EWL). One year after EBT, weight loss differed significantly between good and poor weight-loss-responders (67.5% EWL [95% CI: 64.2, 70.8] vs. 28.2% EWL [95% CI: 25.5, 30.9], *p* < 0.001). Participants who adhered to good lifestyle habits had 4.37 more odds [95% CI: 2.19, 8.88] of being good weight-loss-responders. We also observed that eating four to five meals/day and practicing muscle-strengthening activities >2 times/week were the two lifestyle habits that most significantly determined weight loss response. Furthermore, our results revealed that while adherence to healthy lifestyle habits was a significant determinant of %EWL 3, 6, and 12 months after EBT (*p* < 0.001), emotional eating was a significant determinant of %EWL only 3 and 6 months after the intervention (*p* < 0.01 and *p* < 0.05, respectively). Regarding psychological traits, we observed that neither anxiety nor depression were significant determinants of %EWL. Our results revealed that adherence to healthy lifestyle habits is a significant determinant for weight loss effectiveness among patients with obesity undergoing EBT. These findings highlight the importance of implementing an adequate nutritional intervention program, especially since patients who adhere to good lifestyle habits are able to achieve a weight loss that would be comparable with bariatric surgery.

## 1. Introduction

As obesity has become one of the biggest pandemics of the 21st century, the number of treatment options for obesity has increased significantly [1,2,3]. Still, obesity figures continue to rise, and today, more people are dying from obesity than from being underweight [1]. Obesity treatment options range from lifestyle modification programs to other strategies including pharmacotherapy and bariatric surgery [2,3,4,5]. The latter is known as the most effective modality for long-term weight loss [5]; however, adoption of bariatric surgery is poor due to perceived complications, cost, and fear of undergoing surgery [2,6]. In fact, only 1–2% of the patients eligible for bariatric surgery actually undergo the surgery [6]. Thus, there is a treatment gap for patients with severe obesity who do not want to undergo surgery, who are not candidates for surgery, and even for those patients with moderate obesity (BMI 30–40 kg/m^2^) who cannot lose enough weight through lifestyle changes or pharmacotherapy [2,3,6].

In this context, endoscopic bariatric therapies (EBTs) have emerged as a group of procedures that can bridge the treatment gap between bariatric surgery and non-procedural treatments (e.g., pharmacotherapy and lifestyle therapy) for obesity [2,7,8]. Of the many devices and techniques that constitute EBTs, the intragastric balloon (IGB) and primary obesity surgery endoluminal (POSE) are two procedures that are widely used in the clinical setting. Both EBTs have shifted the treatment paradigm of obesity to less invasive and more cost-effective procedures [2,3,8,9], with the potential to achieve ~40% excess weight loss (EWL) twelve months after the intervention [6,10]—an amount of weight loss that exceeds the threshold set by the American Society for Gastrointestinal Endoscopy and the American Society for Metabolic Bariatric Surgery (AGSE/ASMBS), who establish a minimum of 25% EWL to be clinically significant [10].

Despite the promising results, there is still a great variability in weight loss effectiveness 12 months after EBT interventions [11,12,13,14]. The latter could be attributed to the type of and duration of the EBT as well as the type of nutritional intervention [11,15,16]. In particular, previous research by our group showed that a Mediterranean-style diet plan was more effective for weight loss among these patients [15]. Additionally, Sullivan et al. [11] suggested that the intensity of the nutritional intervention could be another determinant of weight loss in patients submitted to EBTs. Note that an intensive lifestyle program includes three components: a reduced calorie diet, increased physical activity, and behavioral changes that make it easier to adhere to healthy lifestyle habits (e.g., eat fewer calories and become more active) [11,17]. Although this seems intuitive, the synergistic effect between EBTs and the adherence to healthy lifestyle habits on the effectiveness of weight loss has yet to be studied in a clinical setting. Note that in terms of safety and cost effectiveness, EBTs have a clear advantage over bariatric surgery [2,3,6]. Therefore, it is relevant to understand what makes patients undergoing EBTs achieve a weight loss comparable to bariatric surgery. Besides the adherence to healthy lifestyle habits, lessons from lifestyle intervention programs for people with obesity have taught that weight loss success could be linked to eating behaviors (such as emotional eating) and psychological traits (anxiety and depression) [18,19,20,21], all of which are associated with weight loss success, but also with the abandonment (or drop out) of weight loss programs [19,20,21]. Furthermore, a pivotal study by Pontiroli et al. [22] showed that compliance to the rules recommended after bariatric surgery was significantly associated with personality traits such as narcissism. However, little is known about the interaction between such behavioral and psychological traits in patients with obesity undergoing EBTs.

Taking into account the aforementioned, our objective was to investigate the impact of adherence to healthy lifestyle habits on the effectiveness of weight loss in patients submitted to EBTs (IGB or POSE). Considering that EBTs alone exceed the weight loss threshold established by AGSE/ASMBS, we proposed to use the weight loss threshold for bariatric surgery, considering a weight loss of ≥50% EWL as a marker of the weight loss effectiveness [23]. Additionally, our aim was to investigate whether certain eating behaviors and psychological traits could play a role in weight loss effectiveness among these patients.

## 2. Materials and Methods

This prospective longitudinal study included patients with obesity that underwent EBT (IGB or POSE) for weight loss in a private clinic in Barcelona (Spain). Data collection began in August 2018 and lasted until December 2019. Inclusion criteria consisted of: age between 18 to 64 years old, BMI 30–40 kg/m^2^, no previous gastric intervention, no diagnosis of a binge eating disorder or bulimia, and speaking Spanish. According to these criteria, 415 patients were eligible to participate in the study, of whom 408 signed the informed consent and were included in the study (Figure S1). After the baseline visit, 47 participants were excluded (7 subjects because they had an early IGB removal due to intolerance, 20 for lack of compliance to the study protocol since they did not return the questionnaires, and 20 were lost to follow-up), resulting in a final analytical sample of 361 participants. Participants were visited at baseline (before EBT) and then 3, 6, and 12 months after the EBT.

### 2.1. Study Protocol

Recruited participants underwent a 6-month IGB (Medsil^®^ balloon, CSC Medsil, Moskovskaya Oblast, Moscow, Russia), a 12-month IGB (Spatz 3 balloon, Spatz FGIA, Great Neck, New York, NY, USA), or POSE. Note that IGB consists of a space-occupying gastric therapy, in which a saline solution-filled balloon is placed and removed endoscopically after either 6 or 12 months [3,7]. Meanwhile, POSE uses an incision-free operating platform system to create plications in the gastric fundus and body of the stomach, leading to reduced gastric accommodation and delayed gastric emptying [6,7]. Detailed information on IGB and POSE procedures is provided elsewhere [7].

After any of the EBT interventions, participants were advised to comply with different feeding phases as follows: a 3-day progressive clear liquid diet without supplements, followed by a 7-day pureed diet with protein supplements, and 7 days of an easy-to-digest diet. Finally, after 17 days of the EBT intervention, a Mediterranean-style dietary pattern was recommended [24]. Subsequently, all participants received nutritional counseling [17]. Thus, they attended individual sessions of 30–45 min with a registered dietitian every 15 days during the first six months, and monthly afterwards. These sessions included counseling for maintaining healthy lifestyles following the guidelines of the Public Health Agency of Catalonia [24] and Physical Activity for Health from the World Health Organization [25]. Briefly, participants were advised to follow a Mediterranean-style dietary pattern, eat four to five times a day, prioritize home cooking, include plant-based menus for lunch and dinner, and include fresh fruit for dessert. Also, participants were encouraged to drink water instead of other sugary or low-calorie sweetened beverages. Regarding physical activity habits, participants were first advised to practice 150 min/week of moderate physical activity or to perform at least 75 min/week of vigorous physical activity. Once this amount of physical activity was tolerated, participants were advised to engage in 300 min/week of moderate physical activity or to do at least 150 min/week of vigorous physical activity. At that time, participants were also advised to do muscle-strengthening activities 2 or more days/week.

### 2.2. Measurements

#### 2.2.1. Anthropometric Parameters

Wearing light clothing and no shoes, participants were weighed using a scale (Tanita^®^ BC-418, Tokyo, Japan) pre-surgery (baseline) and on each study visit (3rd, 6th and 12th month). Height was measured in meters, without shoes, using a fix wall stadiometer Seca 213 (Hamburg, Germany). This measurement was taken at baseline. We then calculated the body mass index (BMI) as weight (kg) divided by height (m^2^).

#### 2.2.2. Weight Loss Effectiveness

Post-operative weight loss was expressed as the %EWL following the formula: [(initial BMI − post-operative BMI)/(initial BMI) − (ideal weight)] × 100 [26]. Ideal weight was based on a reference body weight of 25 kg/m^2^. In this case, the higher the %EWL, the greater the weight loss effectiveness. Subsequently, weight loss evolution was classified as “good weight-loss-response” (EWL ≥ 50% at nadir and throughout subsequent follow-ups) or “poor weight-loss-response” (EWL < 50% at nadir weight and throughout subsequent follow-ups) [26].

#### 2.2.3. Adherence to Healthy Lifestyle Habits

This variable was assessed through the Eat and Move questionnaire (EMOVE), which was developed to assess the level of adherence to healthy dietary and physical activity habits among patients submitted to EBT [23]. The EMOVE questionnaire was completed by the participants at baseline and on each study visit (3rd, 6th, and 12th month). This questionnaire consists of 15 items, which are rated on a 4-point scale ranging from 0 (“never”) to 3 (“always”). The total score ranges from 0 to 45 points, where higher scores indicate greater adherence to healthy lifestyle habits. In addition, according to the instructions accompanying the EMOVE questionnaire, adherence to healthy lifestyle habits was classified as “poor” (<30 points) or “good” (≥30 points).

#### 2.2.4. Emotional Eating and Psychological Traits

Emotional eating was assessed through the Emotional Eating Questionnaire (EEQ) at baseline [18]. This questionnaire consists of 10 items developed to assess the associations between emotions, eating, and energy intake. All items are rated on a 4-point scale ranging from 0 (“never”) to 3 (“always”). According to the instructions accompanying the EEQ, scores range from 0 to 30; the higher the score, the higher the emotional eating.

Additionally, we evaluated anxiety and depression at baseline with the Symptom Checklist-90 Revised (SCL-90-R) [27]. The SCL-90-R questionnaire consists of 90 items related to the frequency in which certain stressful situations present. All items are rated on a 5-point scale ranging from 0 (“not at all”) to 4 (“extremely”). According to the instructions accompanying the SCL-90-R, from the 90 items, anxiety was evaluated with 10 items and depression with 13 items. In both cases, higher scores are associated to higher anxiety or higher depression symptoms.

### 2.3. Statistical Analyses

Normality was confirmed for all variables using histograms and Q-Q plots. Continuous data are presented as mean ± standard deviation and categorical variables as percentages. General linear models (GLMs) were used to compare %EWL (3, 6, and 12 months) between EBTs. Participants were then classified as poor or good weight-loss-responders according to their %EWL at nadir and subsequent follow-ups. Subsequently, we used GLMs to compare anthropometric parameters, adherence to healthy lifestyle habits (EMOVE questionnaire), emotional eating (EEQ questionnaire), and psychological traits (SCL-90R questionnaire) at baseline between weight loss evolution groups (poor and good weight-loss-responders). Likewise, GLMs were used to compare the %EWL 3, 6, and 12 months after the EBT between weight loss evolution groups. In this case, we also used GLMs to calculate adjusted differences in the %EWL (reference group “good weight-loss-responders”). We also conducted a logistic regression analysis to examine whether subjects were more likely to be good or poor weight-loss-responders 3, 6, and 12 months after the EBT in relation to the level of adherence to a healthy lifestyle (“good” or “poor”). Then, a discriminant function analysis was performed to determine which of the 15 items of the EMOVE questionnaire could reliably classify the subjects as poor or good weight-loss-responders at the beginning (3 months) and at the end (12 months) of the study. Univariate F-tests were then calculated to determine the importance of each independent variable in forming the discriminant functions. Examining the Wilk’s Lambda values for each of the predictors revealed how important the independent variable was to the discriminant function, with smaller values representing greater importance.

Additionally, hierarchical regression analyses were conducted to determine whether the adherence to healthy lifestyle habits, emotional eating, or psychological traits were significant predictors of %EWL 3, 6, and 12 months after the EBTs. In all cases, EMOVE was included in Model 1 as a predictor of %EWL, while Model 2 included EMOVE, emotional eating, anxiety, and depression as predictors of %EWL. 

Finally, hazard ratios (HRs) were calculated to estimate the probabilities of good and poor weight-loss-responders either dropping out or completing the study. In addition, we investigated the differences in %EWL, EMOVE scores, emotional eating, and psychological traits between participants who dropped out or completed the study. All analyses were adjusted for age, gender, initial BMI, and the type of EBT, and performed with the SPSS statistical computer software, version 25.0 (IBM SPSS Statistics, Armonk, NY, USA), except for HRs, which were calculated using the “survival” package in R software, version 3.6.1 (R Foundation for Statistical Computing, Vienna, Austria). Significance testing was considered when *p* < 0.05.

## 3. Results

**Figure 1:**
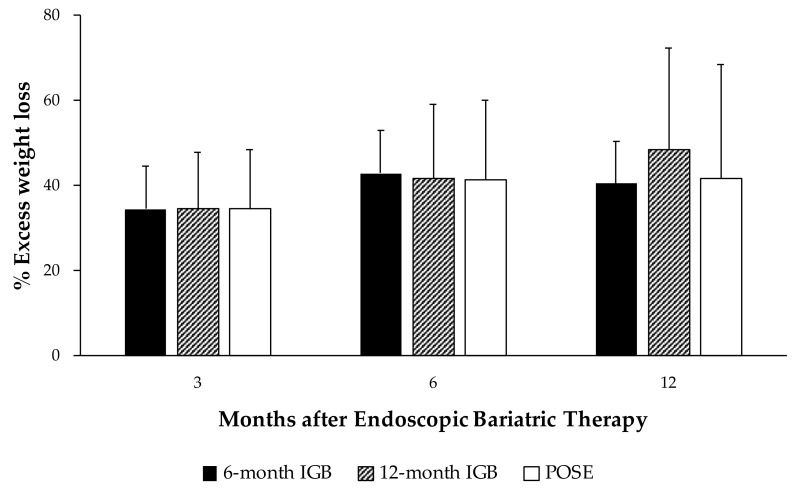
Comparison of excess weight loss between endoscopic bariatric therapies at 3, 6, and 12 months. IGB, Intragastric balloon; POSE, primary obesity surgery endoluminal. Values are expressed as mean and standard error measure. General linear models were used to compare the percentage of excess weight loss between good and poor weight-loss-responders. Analyses were adjusted for age, gender, initial BMI, and type of endoscopic bariatric therapy.

This longitudinal study included 361 patients with obesity (81.4% women; age 41.8 ± 9.5 years; BMI 37.8 ± 4.3 kg/m^2^) who underwent EBTs. Regarding the frequency of EBTs, 24.1% of the participants underwent a 6-month IGB, 46.0% a 12-month IGB, and the remaining 29.9% POSE. Interestingly, our results reveal that the %EWL did not differ between EBTs during the 12 months of follow-up (Figure 1). It is also noteworthy that 3 months after the intervention with EBTs, on average, participants surpassed the minimum threshold of weight loss (>25% EWL) established by the AGSE/ASMBS joint taskforce [10].

Participants were then classified as poor or good weight-loss-responders according to the %EWL at nadir and throughout subsequent follow-ups (Table 1). In this case, we observed that the majority of the population studied (66.5%) were classified as poor weight-loss-responders, while the remaining 33.5% were classified as good weight-loss-responders. As shown in Table 1, no significant differences were found between good and poor weight-loss-responders in anthropometric parameters at baseline. However, we did observe that the EMOVE score at baseline was higher (*p* < 0.05) among good weight-loss-responders, although, on average, both groups had poor adherence to healthy lifestyle habits. Regarding emotional eating and psychological traits, we observed that scores did not differ between good and poor weight-loss-responders.

**Figure 2:**
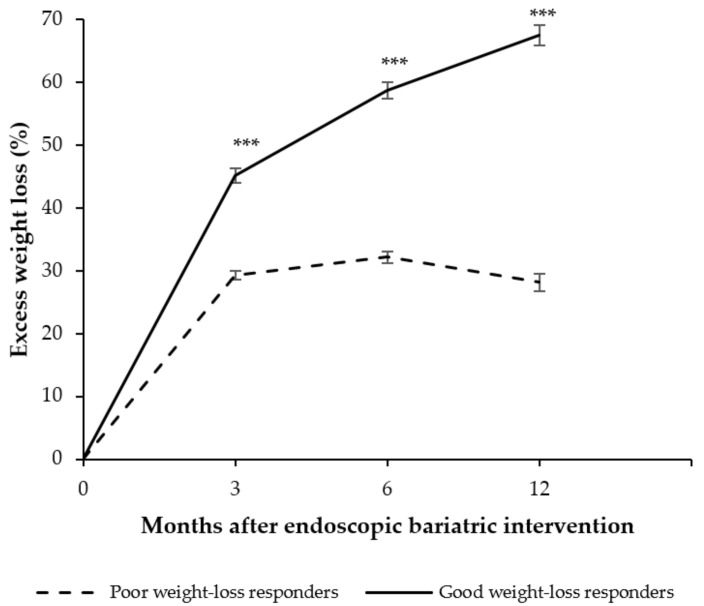
Evolution of weight loss at 3, 6, and 12 months after the endoscopic bariatric therapy. Values are expressed as mean and standard error measure. General linear models were used to compare the percentage of excess weight loss between good and poor weight-loss-responders. Analyses were adjusted for age, gender, initial BMI, and type of endoscopic bariatric therapy. *** *p* < 0.001.

Concerning the evolution of weight loss, we observed that from the 3rd month follow-up %EWL differed significantly between good and poor weight-loss-responders (Figure 2). Accordingly, poor weight-loss-responders lost less %EWL 3, 6 and 12 months after EBT. It is also noteworthy that, relative to the 6th month follow-up, EWL in poor weight-loss-responders was 5.7% lower [95% CI: −7.7, −3.6] at the 12th month follow-up (Figure 2). Meanwhile, good weight-loss-responders had lost 67.5% EWL [95% CI: 64.2, 70.8] at the 12th month follow-up.

### 3.1. Participants with Good Lifestyle Habits Had Higher Odds of Being Good Weight-Loss-Responders

We then quantified the strength of the association between EMOVE categories and weight loss evolution (poor or good weight-loss-responders) using odd ratios (Table 2). Notably, participants who adhered to good lifestyle habits (EMOVE score ≥ 30 points) had significantly higher odds of being good weight-loss-responders at 3, 6, and 12 months after EBTs (Table 2). In addition, participants who adhered to good lifestyle habits at the 12th month follow-up had 4.37 more odds [95% CI: 2.19, 8.88] of being good weight-loss-responders.

### 3.2. Eating Frequency and Practicing Physical Activity Are Lifestyle Habits Associated to a Good Weight Loss Response

Further analyses using a discriminant model showed that 3 months after the EBT, the questions from the EMOVE: ‘*Do you eat between 4 and 5 times a day?*’, ‘*If you eat two dishes, is the second dish smaller than the first?*’, and ‘*Do you practice a minimum of 300 min per week (5 h) of moderate aerobic physical activity or 150 min per week (2.5 h) of vigorous aerobic activity*?’ could classify 65.6% of the cases as either poor or good weight-loss-responders. Meanwhile, 12 months after the EBT, the questions ‘*Do you eat between 4 and 5 times a day?*’ and ‘*Do you perform muscle strengthening activities 2 or more times a week?*’ were the ones that could classify 66.9% of the cases as either poor or good weight-loss-responders.

### 3.3. Adherence to Healthy Lifestyle Habits Is Consistently Associated with %EWL 3, 6, and 12 Months after EBTs

A hierarchical regression analysis showed that 3 months after the EBT (Step 2, Table 3), EMOVE (*β* = 0.60% EWL [95% CI: 0.30; 0.89]) and emotional eating (*β* = 0.48% EWL [95% CI: 0.14; 0.82]) accounted for 12.8% of the variance of %EWL (*p* < 0.001), while neither depression, nor anxiety were significant predictors of %EWL at the 3rd month follow-up. Likewise, 6 months after the EBT (Step 2, Table 3), EMOVE (*β* = 1.04% EWL [95% CI: 0.68; 1.39]) and emotional eating (*β* = 0.49% EWL [95% CI: 0.07; 0.93]) accounted for 20.7% of the variance of %EWL (*p* < 0.001). However, 12 months after the EBT only EMOVE was significantly associated with greater %EWL (1.57 [95% CI: 0.95, 2.18]). Here, we observed that Step 1 (Table 3) accounted for 18.6% of the variance of %EWL (*p* = 0.001).

### 3.4. Poor Weight-Loss-Responders Had Higher Hazard Ratios to Dropping out of the Study

Finally, we observed that compared to good weight-loss-responders, poor weight-loss-responders were more likely to drop out of the study (HR: 2.88 [95% CI: 0.22, 0.53]). This association remained significant after adjusting for age, gender, initial BMI, and type of intervention (HR: 2.96 [95% CI: 0.22, 0.52]). Further analyses revealed that participants who dropped out of the study had lower %EWL (*p* < 0.001) and lower EMOVE scores (*p* < 0.010) 3 and 6 months after the EBT (Table 4). Meanwhile, we observed that neither emotional eating nor the psychological traits differed significantly between participants who completed the study and those who dropped out.

## 4. Discussion

To our knowledge, this is the first study to evidence that patients submitted to EBTs (POSE or IGB) who adhere to healthy lifestyle habits are able to exceed the weight loss threshold of bariatric surgery (which implies that the EWL ≥ 50% at nadir and throughout subsequent follow-ups) [23]. As such, regardless of the type of EBT, participants who adhered to good lifestyle habits during the 12 months after the intervention had 4.37 more odds of being good weight-loss-responders. More interestingly, 12 months after the EBT, good weight-loss-responders had lost ~67.5% EWL, while poor weight-loss-responders gained ~5.7% of the EWL they had lost at the 6th month follow-up. Not surprisingly, compared with good weight-loss-responders, poor weight-loss-responders were more likely to drop out the study (HR: 2.96).

These findings demonstrate that to be successful in facilitating weight loss, EBTs must be used in conjunction with a nutritional intervention that facilitates the adherence to healthy lifestyle habits [6,8,11]. Note that EMOVE score turned out to be a significant determinant of %EWL 3, 6, and 12 months after EBT. This is consistent with previous studies showing that adherence to healthy dietary patterns, rather than restrictive diets, plays a key role in the effectiveness of weight loss among patients undergoing EBTs [15,16]. The latter implies that patients with obesity undergoing EBTs should not simply be on a “diet”; instead, weight loss therapy should include a series of behavioral changes that facilitate healthier eating and becoming more active [17,19].

In line with the aforementioned, we observed that 3 months after the EBT, practicing 300 min/week of moderate physical activity or 150 min/week of vigorous physical activity was associated with a good weight-loss-response. Meanwhile, 12 months after the EBT, the practice of muscle-strengthening activities (≥2 times/week) was the physical activity habit most closely associated with a good weight-loss-response. This is consistent with a systematic review where it was found that people with severe obesity obtained weight loss benefits from both aerobic and strength exercise, probably due to the generation of higher energy expenditure and the stimulation of hypertrophy, understood as the maintenance of muscle mass [28]. Not to mention that physical activity could generate an energy deficit of 500 to 1000 kcal/week, allowing a weight loss of 0.45–0.90 kg/week [28,29].

We also observed that an eating frequency of four to five meals/day was associated with a good weight-loss-response at 3 and 12 months after EBTs. This is noteworthy, as this eating frequency could have a differential effect on metabolic rate, including increased energy expenditure and a greater rate of utilization of fat reserves [30]. Not surprisingly, other epidemiological studies have shown that an eating frequency of four to five meals/day has a positive impact on the prevention of obesity [31,32]. Additionally, previous research performed by our group showed that, relative to an eating frequency of three meals/day, having five meals/day was associated with a lower energy intake between 20:00 and 24:00 [33]. Note that a lower energy intake at night is also associated with lower BMI among people who are overweight and obese [33,34].

Therefore, “practicing muscle-strengthening activities > 2 times/week” and “eating four to five meals/day” are two lifestyle habits that can help patients with obesity after EBT to continue losing weight and adhere to the lifestyle intervention program [19,23]. Especially when patients are struggling to lose weight or maintain the weight loss. Note that 12 months after the EBT, participants who showed a good adherence to healthy lifestyle habits were 337% more likely to be good weight-loss-responders. What is equally interesting is that these patients were able to achieve an amount of weight loss (67.5% EWL) that is comparable to the amount achieved through bariatric surgery [35], suggesting that EBTs in conjunction with the adherence to healthy lifestyle habits offer a clear advantage over bariatric surgery in terms of weight loss effectiveness and cost [2,3,6].

As for the association between emotional eating and weight loss effectiveness, we observed that it was a significant determinant of weight loss at the beginning of treatment and up to the 6th month follow-up. However, this association was lost at the 12th month follow-up. This could be explained by the possibility that emotional people are more prone to engage passionately in a project (such as a lifestyle intervention), but are unable to maintain it for an extended time [36]. This is why, according to Chopra et al. [19], setting realistic goals are also important determinants of weight loss success.

Among other findings, we noted that patients who continued through the 12-month nutritional intervention had greater initial weight loss compared with those who dropped out of the study. Chopra et al. [19] highlighted that greater initial weight loss was considered the most promising predictor of weight loss. According to the authors, early weight loss motivates the patient to adhere to the nutritional intervention program, while building confidence in the intervention and themselves [19]. Note that in our study, poor weight-loss responders were more likely to drop out of the study (HR: 2.96). Interestingly, we observed that neither the emotional eating nor the psychological traits were significantly associated with the dropout rate. This, according to some authors [23,37], could be explained by the fact that patients who are submitted to a bariatric procedure constitute a highly selective group, which results in a homogenized sample of patients, lowering the effect of psychological factors on weight loss outcomes [23].

Our research has certain limitations, starting with the observational nature of the study, which prevents us from claiming causation. Furthermore, we acknowledge as limitations of the study the use of self-reported questionnaires, which are prone to underreporting (i.e., adherence to healthy lifestyle habits) and misreporting (e.g., symptoms of anxiety and depression), and the gender distribution of the sample studied since the proportion of male participants was small.

## 5. Conclusions

In summary, our findings point out that the adherence to healthy lifestyle habits is a key to the weight loss success of EBTs. We would also like to point out that good-weight-loss responders who adhere to good lifestyle habits are able to achieve an amount of weight loss comparable to that which can be achieved with bariatric surgery. Here, we were also able to identify eating frequency and the practice of physical activity as lifestyle habits that could be associated with a good weight-loss-response 3 and 12 months after EBT. Furthermore, we show that poor weight-loss-responders were 2.96 times more likely to drop out of the study. These results emphasize the importance of an adequate nutritional intervention program in patients with obesity undergoing EBTs, since weight loss similar to that of bariatric surgery can be achieved.

## Figures and Tables

**Table 1:** Baseline differences in anthropometric parameters, adherence to healthy lifestyle habits, emotional eating, and psychological traits between poor and good weight-loss-responders.

	Poor Weight Loss-Responders	Good Weight Loss-Responders	*p*-Value
% (*n*)	66.5 (240)	33.5 (121)	
*Anthropometric parameters*			
Weight, kg	101.7 (15.9)	102.0 (17.0)	0.833
Height, m	1.6 (0.1)	1.6 (0.1)	0.870
BMI, kg/m^2^	37.8 (4.2)	37.9 (4.6)	0.675
Fat mass, %	44.8 (5.9)	45.0 (5.7)	0.332
*Adherence to healthy lifestyle habits*			
EMOVE, score	17.9 (6.0)	19.5 (7.2)	**0.036**
*Emotional eating and psychological traits*			
Emotional eating, score	16.4 (6.0)	15.9 (6.4)	0.651
Depression, score	56.3 (10.3)	54.9 (12.1)	0.378
Anxiety, score	54.4 (10.3)	53.0 (10.4)	0.254

BMI: Body mass index; EMOVE, Eat and Move Questionnaire. Data are expressed as mean and standard deviation. General linear models were used to compare anthropometric parameters and determinants of weight loss between poor and good weight-loss-responders. Analyses were adjusted for age, gender, initial BMI, and type of endoscopic bariatric therapy. Significant *p*-values are shown in bold.

**Table 2:** Odd ratios (95% CIs) for weight-loss-response by EMOVE categories.

EMOVE Categories	Weight Loss Effectiveness OR (95% CI)
*3 months*	
Good lifestyle habits	3.23 (1.88; 5.47) ***
Poor lifestyle habits	1 (reference group)
*6 months*	
Good lifestyle habits	3.24 (1.85; 5.67) ***
Poor lifestyle habits	1 (reference group)
*12 months*	
Good lifestyle habits	4.37 (2.19; 8.88) ***
Poor lifestyle habits	1 (reference group)

EMOVE, Eat and Move questionnaire. Table shows odd ratios (OR) and 95% Confidence intervals. Analyses were adjusted for age, gender, initial BMI, and type of EBT. *** *p* < 0.001.

**Table 3:** Hierarchical multivariate regression analyses of predictors of the percentage of excess weight loss (%EWL) 3, 6, and 12 months after the EBT.

Outcome Variable	Predictors	B [95% CI]	R	R^2^	R^2^ Change
*3-month EWL, %*					
*Step 1*			0.303 **	0.092	0.092 **
	EMOVE	0.59 [0.30; 0.89] ***			
*Step 2*			0.358 ***	0.128	0.036 *
	EMOVE	0.60 [0.30; 0.89] ***			
	Emotional eating	0.48 [0.14; 0.82] **			
	Depression traits	−0.07 [−0.33; 0.18]			
	Anxiety traits	0.05 [−0.21; 0.32]			
*6-month EWL, %*					
*Step 1*			0.406 ***	0.165	0.165 ***
	EMOVE	1.03 [0.68; 1.38] ***			
*Step 2*			0.455 ***	0.207	0.042 *
	EMOVE	1.04 [0.68; 1.39] ***			
	Emotional eating	0.49 [0.07; 0.93] *			
	Depression traits	0.23 [−0.12; 0.57]			
	Anxiety traits	−0.14 [−0.50; 0.21]			
*12-month EWL, %*					
*Step 1*			0.431 **	0.186	0.186 **
	EMOVE	1.54 [0.92; 2.15] ***			
*Step 2*			0.459 **	0.211	0.025
	EMOVE	1.57 [0.95; 2.18] ***			
	Emotional eating	0.14 [−0.65; 0.94]			
	Depression	0.52 [−0.08; 1.13]			
	Anxiety	−0.45 [−1.11; 0.20]			

EMOVE, Eat and Move questionnaire; EBT, Endoscopic bariatric therapy. The table shows the unstandardized coefficient (*β*), CI and *p*-value associated with each predictor variable. Analyses were adjusted for age, gender, initial BMI, and type of endoscopic bariatric therapy. * *p* < 0.05; ** *p* < 0.01; *** *p* < 0.001.

**Table 4:** Comparison of studied characteristics between participants who completed the study vs. participants who dropped out.

	Completed	Drop-Out	*p*-Value
% (*n*)	58.2 (210)	41.8 (151)	
*Weight loss evolution*			
3 month EWL, %	33.65 (12.96)	29.53 (14.45)	**<0.001**
6 month EWL, %	41.28 (16.93)	33.98 (18.74)	**<0.001**
*Adherence to a healthy lifestyle*			
Baseline EMOVE, score	18.59 (6.40)	18.61 (6.51)	0.979
3 month EMOVE, score	29.85 (6.13)	27.46 (6.62)	**0.007**
6 month EMOVE, score	29.85 (6.65)	27.20 (6.07)	**0.004**
*Emotional eating and psychological traits*			
Emotional eating, score	16.21 (6.01)	16.54 (6.15)	0.685
Depression, score	56.49 (11.07)	54.96 (11.03)	0.451
Anxiety, score	54.02 (10.27)	53.50 (10.30)	0.942

EMOVE, Eat and Move Questionnaire; EWL, excess weight loss. Data are expressed as mean and standard deviation. General linear models were used to compare anthropometric parameters and determinants of weight loss between poor and good weight-loss-responders. Analyses were adjusted for age, gender, initial BMI, and type of endoscopic bariatric therapy. Significant *p*-values are shown in bold.

## Data Availability

Not applicable.

## Supplementary Materials

The following supporting information can be downloaded at: https://www.mdpi.com/article/10.3390/nu14112261/s1, Figure S1: STROBE diagram of participants flow.
