# Supplementary figures and images for: Adherence to Healthy Lifestyle Habits Is a Determinant of the Effectiveness of Weight Loss among Patients Undergoing Endoscopic Bariatric Therapies
Source: Nutrients. 2022 May 28;14(11):2261. doi: 10.3390/nu14112261 (PMC9183092; doi:10.3390/nu14112261)

**Figure S1.** STROBE diagram of participants flow

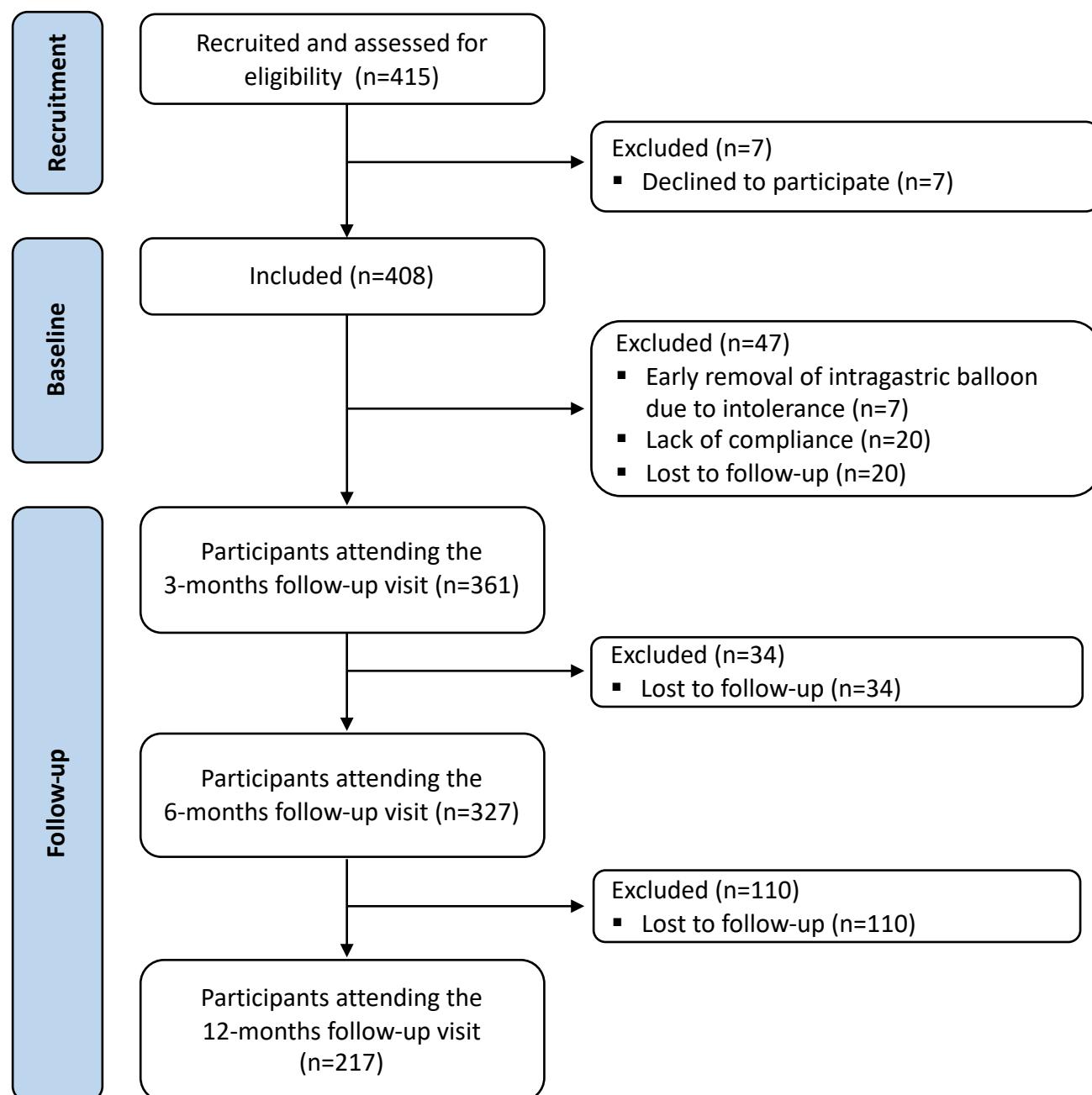

Supplement: Supplementary file 1 [file nutrients-14-02261-s001.zip › nutrients-1737461-supplementary.pdf]
